# mGluR5 receptor availability is associated with lower levels of negative symptoms and better cognition in male patients with chronic schizophrenia

**DOI:** 10.1002/hbm.24976

**Published:** 2020-03-09

**Authors:** Cláudia Régio Brambilla, Tanja Veselinović, Ravichandran Rajkumar, Jörg Mauler, Linda Orth, Andrej Ruch, Shukti Ramkiran, Karsten Heekeren, Wolfram Kawohl, Christine Wyss, Elena Rota Kops, Jürgen Scheins, Lutz Tellmann, Frank Boers, Bernd Neumaier, Johannes Ermert, Hans Herzog, Karl‐Josef Langen, N. Jon Shah, Christoph Lerche, Irene Neuner

**Affiliations:** ^1^ INM‐4, Forschungszentrum Jülich GmbH, Wilhelm‐Johnen‐Straße Institute of Neuroscience and Medicine Jülich Germany; ^2^ Department of Psychiatry, Psychotherapy and Psychosomatics RWTH Aachen University Aachen Germany; ^3^ JARA – BRAIN – Translational Medicine Aachen Germany; ^4^ Department of Psychiatry, Psychotherapy and Psychosomatics University Hospital of Psychiatry Zürich Switzerland; ^5^ INM‐5, Forschungszentrum Jülich GmbH, Wilhelm‐Johnen‐Straße Institute of Neuroscience and Medicine Jülich Germany; ^6^ Department of Nuclear Medicine RWTH Aachen University Aachen Germany; ^7^ INM‐11, Forschungszentrum Jülich GmbH, Wilhelm‐Johnen‐Straße Institute of Neuroscience and Medicine Jülich Germany; ^8^ Department of Neurology RWTH Aachen University Aachen Germany

**Keywords:** chronic schizophrenia, cognition, mGluR5 receptor, negative symptoms, positron emission tomography

## Abstract

Consistent findings postulate disturbed glutamatergic function (more specifically a hypofunction of the ionotropic NMDA receptors) as an important pathophysiologic mechanism in schizophrenia. However, the role of the metabotropic glutamatergic receptors type 5 (mGluR5) in this disease remains unclear. In this study, we investigated their significance (using [^11^C]ABP688) for psychopathology and cognition in male patients with chronic schizophrenia and healthy controls. In the patient group, lower mGluR5 binding potential (BP_ND_) values in the left temporal cortex and caudate were associated with higher general symptom levels (negative and depressive symptoms), lower levels of global functioning and worse cognitive performance. At the same time, in both groups, mGluR5 BP_ND_ were significantly lower in smokers (*F*_[27,1]_ = 15.500; *p* = .001), but without significant differences between the groups. Our findings provide support for the concept that the impaired function of mGluR5 underlies the symptoms of schizophrenia. They further supply a new perspective on the complex relationship between tobacco addiction and schizophrenia by identifying glutamatergic neurotransmission—in particularly mGluR5—as a possible connection to a shared vulnerability.

## 1 INTRODUCTION

More than 100 years after the implementation of the schizophrenia concept, this severe illness remains one of the most mysterious and costliest mental disorders in terms of human suffering and social expenditure, with a lifetime prevalence of about 1% worldwide (van Os & Kapur, 2009). Numerous genetic and environmental factors contribute to the development of schizophrenia (Howes, McCutcheon, Owen, & Murray, 2017). On a neurobiological level, a coherent body of evidence attributes a prominent role to the dysfunctions of dopaminergic neurotransmission, although pharmacological and other studies show that dopaminergic dysfunction is unlikely to explain the full range associated to the disorder (Winton‐Brown, Fusar‐Poli, Ungless, & Howes, 2014).

Complementary evidence from pharmacology, physiology, and brain imaging suggest that glutamatergic dysfunction might contribute to the biological processes underlying some of the clinical features in schizophrenia (Moghaddam & Javitt, 2012), and this has been further supported by multiple animal, post‐mortem and genetic studies (Egerton & Stone, 2012; Hammond, Shan, Meador‐Woodruff, & McCullumsmith, 2014; Javitt, 2010). These investigations implicate a complex deregulation of glutamatergic neurotransmission, not solely limited to a simple deficit in glutamatergic neurotransmission but rather mainly associated with aberrant glutamate receptor function and localization (Hammond et al., 2014).

In particular, there are consistent findings which imply that schizophrenia is primarily associated with the hypofunction of *N*‐methyl‐d‐aspartate glutamate receptors (NMDA‐R), leading to a hypostimulation of gamma‐amino‐butyric‐acid (GABA) inhibitory interneurons and disinhibited glutamate release (Egerton & Stone, 2012). The evidence for this conclusion originates from the psychosis model experimentally induced by noncompetitive NMDA‐R antagonists, such as phencyclidine (PCP) and ketamine (Abi‐Saab, D'Souza, Moghaddam, & Krystal, 1998). However, in vivo investigation of NMDA‐R is difficult due to the high nonspecific binding and poor signal of the available NMDA‐R radioligands (Waterhouse, 2003). To date, there has only been one published SPECT study of NMDA‐R binding in schizophrenia patients that confirms a relative deficit in NMDA‐R activity in the left hippocampus in unmedicated subjects (Pilowsky et al., 2006).

New Positron emission tomography (PET) radiotracers allow the investigation of another type of receptor closely associated with NMDA‐R—the metabotropic glutamatergic receptors type 5 (mGluR5). The mGluR5 receptors are G‐protein coupled receptors abundantly expressed on cortical, hippocampal and striatal regions in the human brain and are highly implicated in the pathophysiology of schizophrenia (Matosin, Fernandez‐Enright, Lum, & Newell, 2017). The mGluR5 receptors share a structural and functional link with the NMDA‐R (Newell & Matosin, 2014) and jointly modulate glutamatergic signaling (Newell, 2013). The activation of mGluR5 results in an enhanced functionality of the NMDA‐R (Niswender & Conn, 2010). In this connection, it has been shown that a mGluR5 agonist effectively reverses the (pro‐psychotic) behavioral effects of ketamine (Chan, Chiu, Sou, & Chen, 2008), indicating, once again, that mGluR5 plays an important role in the regulation of NMDA‐mediated neurotransmission in schizophrenia‐related processes.

To date, a genetic linkage study (Volk, Eggan, & Lewis, 2010) and, more significantly, studies from animal experiments have provided conclusive evidence for mGlu5 receptors playing a significant role in the pathogenesis of schizophrenia. An investigation has demonstrated deficiencies in NMDA‐dependent plasticity and learning behaviors in mGluR5 knockout mice (Lu et al., 1997). Furthermore, mGluR5 knockout animals exhibit deficits in prepulse inhibition, a measure of sensorimotor gating, which is impaired in schizophrenia patients and can be reversed by antipsychotic agents (Chen, Stoker, & Markou, 2010). Moreover, mGluR5 antagonists have been shown to induce locomotor hyperactivity, prepulse inhibition (PPI) deficits, and learning impairments (Campbell et al., 2004; Homayoun, Stefani, Adams, Tamagan, & Moghaddam, 2004; Kinney et al., 2003; Pietraszek et al., 2005). On the other hand, it has been shown that mGluR5 activation ameliorated cognitive deficits in several animal models (Balschun, Zuschratter, & Wetzel, 2006). It has also been demonstrated that mGluR5 plays an important role in the induction of long‐lasting forms of synaptic plasticity, including long‐term depression (LTD) and long‐term potentiation (LTP) of transmission at multiple glutamatergic synapses and also induces long‐lasting changes in neuronal excitability (Kullmann & Lamsa, 2008). Overall, the animal studies referred to here emphasized that a decreased availability (or/and function) in mGluR5 may be involved in the symptomatology of schizophrenia.

Taken together these findings have directed attention toward a possible therapeutic modification of the symptoms of schizophrenia via mGluR5. Indeed, positive allosteric modulators (PAM) of mGluR5 receptors revealed some promising effects. These drugs act by binding to the allosteric site on the mGluR5 to potentiate its activation and subsequently the activation of NMDA‐R by glutamate (Chan et al., 2008; Niswender & Conn, 2010). Thereby, they show some utility as novel antipsychotic and cognition‐enhancing drugs with efficacy against all three symptom‐domains (positive, negative, and cognitive; Maksymetz, Moran, & Conn, 2017). In addition, several recent animal studies have confirmed the pro‐cognitive effects of PAM (Golubeva, Moloney, O'Connor, Dinan, & Cryan, 2016).

However, the success of new mGluR5‐based therapies depends on a deeper understanding of the role of mGluR5 in schizophrenia and other brain disorders.

The recently developed radiotracer [^11^C]ABP688 (3‐(6‐methyl‐pyridine‐2‐ylethynyl)‐cyclohex‐2‐enone‐O‐[^11^C]‐methyloxime) has opened up new possibilities to evaluate mGluR5 in PET studies as it binds to the allosteric site of the mGluR5 and is not able to bind glutamate (Ametamey et al., 2007). Previous investigations have shown gender differences (Smart et al., 2019) and a sufficient test–retest reliability for this radiotracer (Smart et al., 2018) and confirmed its ability to detect physiological changes in endogenous glutamate levels (DeLorenzo, Kumar, Mann, & Parsey, 2011).

[^11^C]ABP688 has previously been used to investigate several psychiatric illnesses and repeated intraindividual measures using [^11^C]ABP688 have been able to show a reduction in mGluR5 availability following the administration of ketamine in patients with depression and healthy volunteers (DeLorenzo et al., 2015; Esterlis et al., 2018). Furthermore, an association between lower mGluR5 availability and related functional connectivity alterations has been reported in drug‐naïve young adults with major depression without comorbidity (Kim et al., 2019). Moreover, altered mGluR5 availability was shown in the temporal lobe and amygdala in patients with an alcohol disorder (Akkus et al., 2018) and obsessive–compulsive disorder (Akkus et al., 2014).

Only a small number of studies investigated the role of mGluR5 receptors in schizophrenia. Akkus et al. (2017) did not find differences in mGluR5 distribution volume ratio (DVR) between controls and patients but found a correlation between DVR in the medial orbitofrontal cortex and the use of antipsychotics. A highly significant global reduction in mGluR5 density has been reported in smokers and also in ex‐smokers and is thought to be a possible result of an adaptation to a chronic increase in glutamate, induced by chronic nicotine administration (Akkus et al., 2013; Akkus et al., 2016; Hulka et al., 2014). Bearing in mind the very high prevalence of smoking (more than 60%) in subjects with schizophrenia compared to the general population (de Leon & Diaz, 2005; Sagud et al., 2009), the possible mediating role of mGluR5 as a linkage between schizophrenia and smoking (tobacco misuse or addiction) requires a closer investigation. Indeed, patients with schizophrenia and comorbid nicotine dependence are more likely to have an earlier age of onset of the disease, higher symptom severity as expressed by the Positive and Negative Syndrome Scale (PANSS; Schwartz et al., 2005) and a lower school performance (Riala et al., 2005) than nonsmoking patients. However, it is still unclear whether the neurobiology of schizophrenia makes patients more vulnerable to the addiction or whether nicotine is used with the aim to “recover” cognitive deficits (De Luca et al., 2004).

The aim of our exploratory study was to investigate the role of mGluR5 receptors for psychopathology and cognition in patients suffering from schizophrenia. Taking into consideration the association between NMDA‐R and mGluR5 receptors, we hypothesized that a higher availability of mGluR5 receptors will be advantageous for better cognition and less pronounced psychopathology in schizophrenia patients due to the modulatory effects on NMDA‐R. Furthermore, we expected a general reduction of mGluR5 receptor binding in schizophrenia patients compared to healthy volunteers. In accordance with previous studies, we also expected a strong reduction in receptor binding due to smoking status in both groups.

## 2 MATERIALS AND METHODS

### 2.1 Participants

The sample consists of 15 male patients diagnosed with schizophrenia and 15 healthy male volunteers matched for age, smoking status, gender, and education. Both groups had an average age of 40.20 ± 10.40 and 41.60 ± 10.80 years, respectively. Subjects were instructed not to drink coffee or alcohol and not to take any medicine within 24 hr before the PET measurement (except their daily medication). Smokers were not allowed to smoke for 2 hr prior to injection of the tracer.

All patients were treated with second‐generation antipsychotics (mean chlorpromazine equivalents (Andreasen, Pressler, Nopoulos, Miller, & Ho, 2010): 277.8 ± 195.1 mg/day (ranging between 94.3 and 683.8 mg/day); mean olanzapine equivalents according to the classical mean doses method (Leucht et al., 2015) 17.2 ± 9.8 mg (ranging between 5 and 37.15 mg). Ten patients were treated mono‐therapeutically, while five received two antipsychotics: Amisulpride and clozapine (3), aripiprazole and clozapine (1), or quetiapine and sertindole (1).

The study was approved by the Ethics Committee of the Medical Faculty at the RWTH Aachen University and the German Federal Office for Radiation Protection (*Bundesamt für Strahlenschutz*). Patients were recruited from Uniklinik RWTH Aachen and complied to the diagnostic criteria according to DSM‐IV (First, Spitzer, Gibbon, & Williams, 2002). Informed consent was obtained for all subjects.

### 2.2 General psychopathology and functioning assessment

The general psychopathology in patients suffering from schizophrenia was assessed using the PANSS (Kay, Fiszbein, & Opler, 1987). The analysis was performed according to the traditional three‐factorial structure. For a more precise assessment of the negative symptoms, we used the Scale for the Assessment of Negative Symptoms (SANS; Andreasen, 1982). The scale consists of five subscales that evaluate five different domains of negative symptoms: Affective flattening, alogia, avolition‐apathy, anhedonia‐asociality, and attentional impairment. Each domain is split into observable behavioral symptoms and a global level. These are rated on a scale from 0 to 5. Higher scores describe more pronounced symptoms. For the assessment of depressive symptoms, we used the Hamilton Depression Scale (HAMD) with 21 items (Hamilton, 1960). The general functioning level was assessed using the Global Assessment of Functioning (GAF; Piersma & Boes, 1997), constructed as an overall (global) measure of how patients are doing. GAF rates psychological, social, and occupational functioning, covering the range from positive mental health to severe psychopathology (Aas, 2011). The 100‐point scales of GAF are divided into intervals, each with 10 points (for example 31–40 and 51–60). The 10‐points intervals have anchor points (verbal instructions) describing symptoms and functioning that are relevant for scoring. The anchor points for interval 1–10 describe the most severely ill, and the anchor points for interval 91–100 describe the healthiest (Aas, 2010). We used the German version of the scale (American Psychological Association, 2003). The assessment of social and occupational functioning using the Global Functioning: Social (GF: Social; Auther, Smith, & Cornblatt, 2006) and the Global Functioning: Role (GF: Role; Niendam, Bearden, Johnson, & Cannon, 2006) scales. The scales have been partially derived from Social and Occupational Functioning Assessment Scale (SOFAS) from the Diagnostic and Statistical Manual of Mental Disorders, Fourth Edition (DSM‐IV) and the GAF, as it appears in the Scale of Prodromal Symptoms (SOPS; Cornblatt et al., 2007). They represent parallel (one targeting social, the other role) well‐anchored scales that take age and phase of illness into account. The functioning level of the patients is rated during a structured interview, ranging between 1 (“extreme social isolation”/“extreme role dysfunction”) and 10 (“superior social/interpersonal functioning”/“superior role functioning”).

### 2.3 Neurocognitive tests

The neuropsychological test battery consisted of several tests, chosen according to the MATRICS Consensus Cognitive Battery (Nuechterlein et al., 2008). The focus in our examination was on three cognitive domains, recently shown to explain about 86% of the variance for the composed cognitive score in stable patients with schizophrenia (Georgiades et al., 2017): Speed of processing (Trail Making Test, Part A, the coding task from the Wechsler Intelligence Scale [WAIS‐IV]), working memory (Letter‐Number Span test and Corsi block‐tapping task) and visual learning (Rey Visual Design Learning Test).

#### 2.3.1 Trail‐making test

The Trail‐Making Test (TMT) is a popular test because of its high sensitivity to the presence of cognitive impairment (Kortte, Horner, & Windham, 2002). The test is divided into two parts. In Part A, the participant is required to draw a line connecting 25 numbers consecutively as quickly as possible. In Part B, the participant must draw a line alternating between numbers and letters in consecutive order. Performance is assessed by the time taken to complete each trial correctly. The TMT reflects a combination of several cognitive functions, measuring complex visual scanning with a motor component, motor speed, and agility. Part B is particularly sensitive to cognitive flexibility and executive function. The difference score TMT(B)–TMT(A) and the ratio score TMT(B)/TMT(A) are reported to be more indicative for executive control abilities (Sanchez‐Cubillo et al., 2009) and an index of cognitive flexibility, relatively independent of manual dexterity (Vazzana et al., 2010).

#### 2.3.2 Number‐symbol coding task

The number‐symbol coding task is a subtest of the revised Wechsler Intelligence Scale (WAIS‐IV; Lawrence, Saklofske, Coalson, & Raiford, 2010). Participants are asked to pair symbols to numbers and write them into blank squares as quickly as possible, referring to a digit symbol key outlined at the top of the examination sheet. Scoring is based on the number of correct substitutions made in 120 s. It primarily quantifies the speed of processing but also quantifies short‐term visual memory, learning ability, cognitive flexibility, attention, concentration, and motivation.

#### 2.3.3 Letter‐number span

In the Letter–Number Span (LNS; Gold, Carpenter, Randolph, Goldberg, & Weinberger, 1997), the tester verbally presents increasingly longer sequences of intermixed numbers and letters at a rate of one per second. After each sequence, the participant is asked to repeat the numbers in ascending order first, and then the letters in alphabetical order. The letter–number sequences range from two up to a maximum length of seven stimuli. Four trials are presented for each length of the sequence. The test is discontinued when the subject fails four consecutive trials of the same length. One point is scored for each correctly repeated sequence (maximum total score is 24 points). This test measures the performance of the working memory.

#### 2.3.4 Corsi block‐tapping task

The Corsi block‐tapping task (Corsi, 1973) is a simple and powerful test used for the assessment of deficits in immediate nonverbal memory and for clarifying theoretical conceptions of visuospatial memory (Berch, Krikorian, & Huha, 1998). The original Corsi apparatus consisted of a set of nine identical blocks (3 × 3 × 3 cm) irregularly positioned on a wooden board (23 × 28 cm). The experimenter points to a series of blocks at a rate of one block per second. Subsequently, the participant is required to point to the same blocks in their order of presentation. The length of the block sequences increases until recall is no longer correct (Vandierendonck, Kemps, Fastame, & Szmalec, 2004). The final test scores represent a sum of correct recalls performed forward and backward.

#### 2.3.5 Rey visual design learning test

The Rey visual design learning test (RVDLT; Strauss, Sherman, & Spreen, 2006) is an instrument that assesses immediate memory span, new learning, and recognition memory for nonverbal material (Wilhelm, 2004). Fifteen cards with geometric figures are presented over five learning trials, with immediate recall tested after each presentation. The total score is the number of correctly memorized items reproduced by means of drawings in the trials and a delayed recall test. In the recognition test, subjects are asked to pick the target items from a set of 30 items, which contains 15 targets and 15 nontargets.

#### 2.3.6 Z‐composite score

For additional analysis, we calculated the summarized z‐scores for all cognitive parameters. Here, the subscores from all of the tests were transformed such that higher values consistently corresponded to better cognitive performance. These transformed measures were then converted to standardized scores by setting the sample mean of each measure to zero and the standard deviation to 1. A composite z‐score was then computed as the mean score of the sum of the several standardized scores.

### 2.4 Positron emission tomography

Radiosynthesis of [^11^C]ABP688 was performed according to the method already reported (Elmenhorst et al., 2016). The average molar activity at the injection time was 101.70 ± 45.33 MBq/nmol. All subjects were measured using a 3 T hybrid MR‐BrainPET insert system (Herzog et al., 2011). Our bolus plus infusion protocol was optimized based on a previous publication that reported a *K*_bolus_ = 53 min and a steady‐state after 40 min (Burger et al., 2010). In our protocol, the bolus injection (50% of total activity), followed by 65 min of infusion with 92 ml/hr infusion rate, was applied after positioning the subject in the scanner. The average total injected activity per subject was 525 ± 55 MBq. A distribution equilibrium was observed after 30 min (also reported by other researchers; Akkus et al., 2014; Deschwanden et al., 2011). Starting simultaneously with the bolus injection, the PET data were acquired in list mode for 65 min. The image reconstruction was performed with 3D‐OP‐OSEM (van Velden et al., 2008; 2 subsets, 32 iterations), with an isotropic voxel of 1.25 mm into a volume consisting of 153 transverse slices of 256 × 256 pixels, and a frame scheme of 10 × 60 s; 11 × 300 s. The images were corrected for attenuation (Rota Kops, Herzog, & Shah, 2014), random and scattered coincidences, and dead time. Postprocessing was performed first by filtering with a 2.5 mm 3D Gaussian filter and then by performing motion correction with frame‐by‐frame realignment. Herby, the reference image was obtained, on average, at 5 min postinjection (Scheins et al., 2019 2019).

PMOD software version 3.9 was used to define the volumes of interest (VOIs) with T1 MPRAGE images serving as the anatomical reference. All images were normalized to the MNI space, and the Hammers atlas (Hammers et al., 2003) was used for activity concentration analysis. The maximum probability operation was applied to generate the VOIs related to the gray matter cortex (GM) in all brain temporofrontal regions except in basal ganglia regions. The cerebellum GM has been chosen as the most appropriate reference region for human brain studies on mGluR5 (Akkus et al., 2017; Akkus et al., 2018; Smart et al., 2018). In all cases, the nondisplaceable binding potential (BP_ND_) of [^11^C]ABP688 was estimated as:

$${\mathrm{BP}}_{\mathrm{ND}}=\left[\frac{C_{\text{region}}}{C_{\mathrm{ref}}}-1\right],$$

where *C*_region_ is the specifically bound radioligand concentration and *C*_ref_ is the nondisplaceable ligand concentration in the cerebellar GM reference tissue. BP_ND_ values were calculated in an average of 5 min frame after 30 min p.i during the equilibrium plateau.

### 2.5 Statistical analysis

Statistical analyses were performed using the Statistical Package 25.0 (IBM SPSS Inc., Chicago, IL). We compared the neurocognitive performance between the groups using Student's *t*‐tests. The relationship between the mGluR5 receptor binding (expressed as BP_ND_ values) and psychopathology, as well as neurocognitive performance (in case of neurocognitive performance separately for each group), was exploratory examined by calculating the Spearman's rank correlation coefficient between scores reached in the neurocognitive tests and BP_ND_ in seven observed brain regions. We calculated r‐squared values (*r* ^2^) to evaluate the goodness‐of‐fits and to determine what fraction of the variability of the independent variable is explained by particularly independent variables. In all tests, values of *p* < .05 were considered statistically significant. All tests were two‐tailed. Missing values were not replaced. For the analysis of the group effects, BP_ND_ values were applied in general linear models, including all analyzed brain regions as a repeated variable, groups and smoking status as factors (between‐subject effects) and age as a covariate. Corrections for multiple comparisons using the Bonferroni method were applied and updated *p* values were reported.

## 3 RESULTS

### 3.1 General psychopathology

The general sociodemographic properties are given in Table 1. The psychopathological properties of the patient sample, as well as separately between subgroups with different smoking statuses, are given in Table 2. The scores were compared between the subgroups using the nonparametric Wilcoxon test. The “smokers” subgroup had higher scores in all tests rating the general psychopathology (PANSS, SANS, and HAMD).

**Table 1:** Baseline demographics and clinical characteristics of patients and healthy volunteers

	Patients (*N* = 15)	Healthy controls (*N* = 15)
Age	40.33 ± 11.07	41.67 ± 11.15
Ethnic background		
Caucasian	12 (80%)	15 (100%)
African	1 (6.7%)	0 (0%)
Arabian	2 (13.3%)	0 (0%)
Years of education	11.00 ± 1.46	11.73 ± 1.49
Estimated IQ (WST)	103.53 ± 12.81	111.93 ± 25.64
BMI	28.75 ± 4.13	24.03 ± 7.44
Smoking status		
Yes	9 (60%)	8 (53.3%)
No	6 (40%)	7 (46.7%)
Mean illness duration (years)	19.00 ± 11.02	–
Diagnosis (ICD‐10)		
F20.0	11 (73.3%)	–
F20.3	1 (6.7%)	–
F20.6	2 (13.3%)	–
F20.9	1 (6.7%)	–

*Note*: Data are given as mean ± *SD*, or *n*/*N* (%).

Abbreviations: BMI, body‐mass index; F20.0, Paranoid Schizophrenia; F20.3, Undifferentiated Schizophrenia; F20.6, Simple Schizophrenia; F20.9, Schizophrenia, Unspecified; WST, Wortschatztest (vocabulary test; Klaus‐Helmut et al.1992).

**Table 2:** Baseline clinical assessment scores in the patient group

	Patients (*N* = 15)	Smokers (*N* = 9)	Nonsmokers (*N* = 7)	Smokers versus nonsmokers (*p* values)
*Basic assessment scores*				
PANSS	68.20 ± 9.38	72.0 ± 10.30	62.50 ± 3.30	.057
PANSS positive	15.60 ± 2.35	16.80 ± 2.70	14.83 ± 1.60	.388
PANSS negative	17.07 ± 4.43	19.00 ± 4.90	14.20 ± 0.40	.026
PANSS general score	35.53 ± 4.24	36.90 ± 4.90	33.50 ± 1.90	.181
SANS: Total score	17.60 ± 19.43	26.70 ± 20.30	4.00 ± 5.10	.008*
SANS: Affective flattening	2.73 ± 4.83	4.40 ± 5.70	0.20 ± 0.40	.145
SANS: Alogia	1.13 ± 1.92	1.90 ± 2.20	0.00 ± 0.00	.088
SANS: Avolition	3.53 ± 3.58	5.30 ± 3.60	0.80 ± 0.80	.088
SANS: Anhedonia	6.93 ± 6.68	10.00 ± 6.50	2.30 ± 3.80	.026
SANS: Attention impairment	3.37 ± 4.37	5.00 ± 4.90	0.70 ± 1.20	.026
HAMD	5.80 ± 6.61	9.10 ± 6.70	0.80 ± 1.20	<.001*
GAF	64.67 ± 17.12	54.30 ± 11.90	80.20 ± 10.50	.002*
GF: Social	7.43 ± 1.60	6.40 ± 0.90	8.80 ± 1.20	.003*
GF: Role	5.79 ± 2.89	3.80 ± 1.90	8.50 ± 0.80	.001*

*Notes*: Scores were further depicted for smokers and nonsmokers separately and compared between the subgroups using the nonparametric Wilcoxon test. *Significant results after applying Bonferroni correction (*p* < .0083). Values are given as mean ± *SD*.

Abbreviations: GAF, Global Functioning Scale; GF: Role, Global Functioning: Role; GF: Social, Global Functioning: Social; HAMD, Hamilton Depression Rating Scale; PANSS, Positive and Negative Syndrome Scale; SANS, Scale for the Assessment of Negative Symptoms; SHAPS, Snaith–Hamilton Pleasure Scale.

In particular, the comparison between the subgroups regarding the SANS total score (*p* = .008) and the HAMD score (*p* < .001) remained significant after Bonferroni correction (corrected *p*‐value for six different psychopathological tests: *p* < .0083). Both test procedures for the assessment of global functioning (GAF and GF) revealed significantly higher values in nonsmoker than smoker subjects in terms of higher functioning levels (GAF: *p* = .002, GF social: *p* = .003, and GF Role: *p* = .001).

### 3.2 BP_ND_ analysis

**Figure 1:**
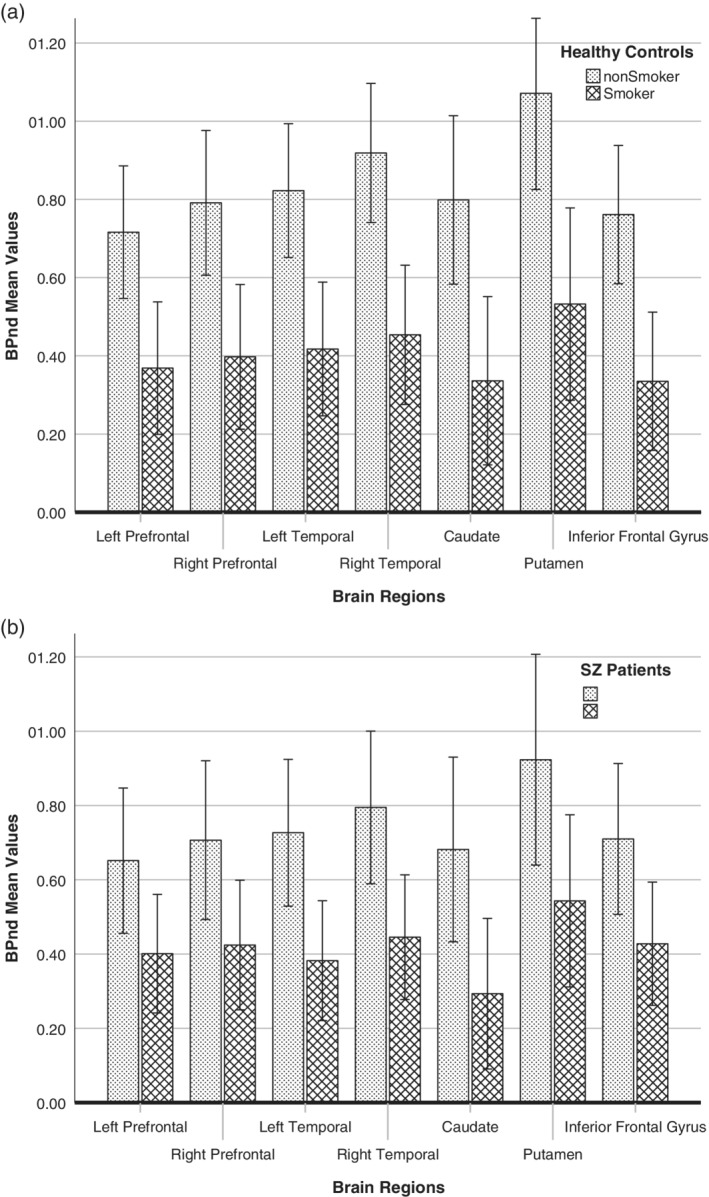
(a) Mean BP_ND_ values in seven regions of the brain in healthy controls according to smoking status, and (b) Mean BP_ND_ values in seven regions of the brain in schizophrenia patients according to smoking status (error bars are ±2 *SEM*)

**Figure 2:**
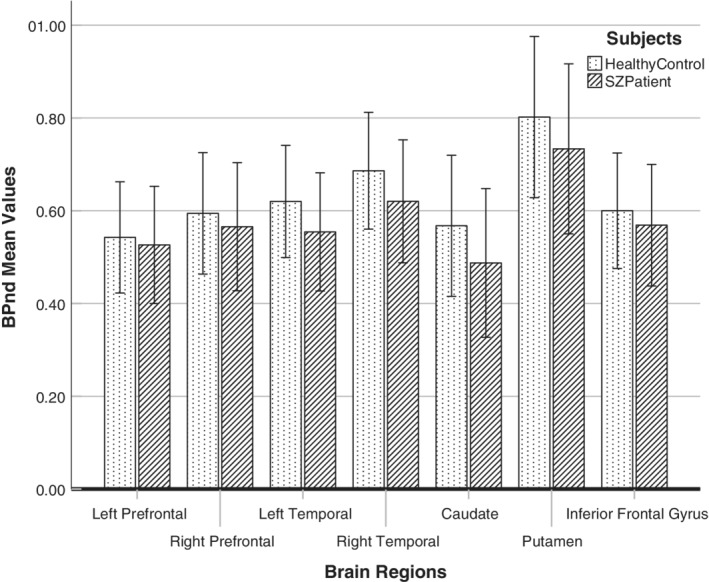
Comparison between mean BP_ND_ values in seven regions of the brain for healthy controls and patients with schizophrenia (error bars are ±2 *SEM*)

No significant differences in BP_ND_ were observed between healthy controls and schizophrenia patients, when considering all of the observed brain regions (*F*_[27,1]_ = 0.285; *p* = .598; effect η_p_^2^ = 0.010). Age as a covariate was not significant in the BP_ND_ general linear model analysis (*p* > .05). The BP_ND_ did not correlate with the medication intake (chlorpromazine equivalents) and illness duration in any region. However, we observed, as others (Akkus et al., 2013; Akkus et al., 2016; Akkus et al., 2017), a significant difference between smokers and nonsmokers *F*_(27,1)_ = 15.500; *p* = .001; effect η_p_^2^ = 0.365, and a reduction of up to 50% in BP_ND_ values in smokers_,_ according to the observed brain region (Figure 1a,b). Figure 2 shows the BP_ND_ comparison between groups, but this was not found to be significant. The BP_ND_ values between the groups and according to smoking status are presented in Table 3.

**Table 3:** BP_ND_ values, comparison between smokers and nonsmokers subgroups and comparison between smoker/nonsmoker considering both groups (evaluated by repeated measures general linear models including all regions in the analysis with groups and smoking status as factors)

	Patients			Healthy controls		
	Smokers (*N* = 9)	Nonsmokers (*N* = 6)	Smokers/nonsmokers (*N* = 15)	Smokers (*N* = 8)	Nonsmokers (*N* = 7)	Smokers/nonsmokers (*N* = 15)
Prefrontal left	0.40 ± 0.08	0.65 ± 0.09	0.62 ± 0.09	0.37 ± 0.06	0.72 ± 0.11	0.51 ± 0.13
Prefrontal right	0.42 ± 0.08	0.71 ± 0.09	0.59 ± 0.09	0.40 ± 0.06	0.79 ± 0.12	0.51 ± 0.13
Temporal left	0.38 ± 0.07	0.73 ± 0.08	0.52 ± 0.08	0.42 ± 0.07	0.82 ± 0.12	0.51 ± 0.14
Temporal right	0.44 ± 0.07	0.79 ± 0.08	0.56 ± 0.08	0.45 ± 0.07	0.92 ± 0.12	0.49 ± 0.14
Caudate	0.29 ± 0.11	0.68 ± 0.07	0.43 ± 0.11	0.34 ± 0.08	0.80 ± 0.13	0.42 ± 0.15
Putamen	0.54 ± 0.11	0.92 ± 0.09	0.58 ± 0.12	0.53 ± 0.09	1.07 ± 0.17	0.50 ± 0.12
Inferior frontal gyrus	0.43 ± 0.08	0.71 ± 0.09	0.60 ± 0.09	0.42 ± 0.06	0.78 ± 0.12	0.54 ± 0.13

*Note*: Nondisplaceable binding potential (BP_ND_) values are represented by mean ± *SEM*.

### 3.3 Relationship between general psychopathology and mGluR5 receptor binding

The relationship between general psychopathology and mGluR5 receptor binding was examined by calculating a Spearman's rank correlation coefficient between scores reached in the tests for psychopathological assessment and BP_ND_ in the seven observed brain regions (prefrontal cortex right and left; temporal cortex right and left; caudate; putamen and inferior frontal gyrus). These regions were selected based on our previous literature review of schizophrenia research (Corti et al., 2011; Fatemi, Folsom, Rooney, & Thuras, 2013; Gupta et al., 2005; Ohnuma, Augood, Arai, McKenna, & Emson, 1998; Volk et al., 2010) relating to frequently involved regions and were also based on studies which used [^11^C]ABP688 to show higher uptake (mGluR5 receptor density) in the selected regions (Akkus et al., 2017; Ametamey et al., 2007). The correlations are given in Table 4.

**Table 4:** Relationship between the general psychopathology and mGluR5 receptor binding in SZ patients examined by calculating Spearman's rank correlation coefficient between scores reached in the tests for psychopathological assessment and BP_ND_ values in the seven observed brain regions

		Left prefrontal gyrus	Right prefrontal gyrus	Left temporal	Right temporal	Caudate	Putamen	Inferior frontal gyrus
Subjects	*N*	15	15	15	15	15	15	15
PANSS positive	*r*	−.488	−.494	−.431	−.469	−.057	−.492	−.522
	*p*	.065	.061	.109	.078	.025	.062	.046
PANSS negative	*r*	−.473	−.535	−.714*	−.610	−.679*	−.539	−.639
	*p*	.075	.04	.003*	.016	.005*	.038	.010
PANSS general	*r*	−.638	−.679*	−.665*	−.596	−.803*	−.612	−.665*
	*p*	.01	.005*	.007*	.019	.000*	.015	.007*
PANSS total	*r*	−.632	−.668*	−.734*	−.669*	−.793*	−.621	−.727*
	*p*	.011	.007*	.002*	.006*	.000*	.013	.002*
SANS: Affective flattening	*r*	−.612	−.608	−.669*	−.563	−.631	−.500	−.660*
	*p*	.015	.016	.006*	.029	.012	.058	.007*
SANS: Alogia	*r*	−.486	−.541	−.690*	−.561	−.708*	−.475	−.618
	*p*	.066	.037	.004*	.029	.003*	.074	.014
SANS: Avolition‐apathy	*r*	−.644	−.687*	−.792*	−.714*	−.762*	−.687*	−.773*
	*p*	.01	.005*	.000*	.003*	.001*	.005*	.001*
SANS: Anhedonia	*r*	−.208	−.293	−.482	−.315	−.533	−.177	−.278
	*p*	.457	.289	.069	.252	.041	.529	.315
SANS: Attentional impairment	*r*	−.343	−.426	−.549	−.384	−.600	−.343	−.413
	*p*	.210	.114	.034	.158	.018	.210	.126
SANS: Global score	*r*	−.411	−.503	−.620	−.463	−.715*	−.401	−.498
	*p*	.128	.056	.014	.082	.003*	.138	.059
HAMD	*r*	−.369	−.428	−.590	−.455	−.663*	−.430	−.456
	*p*	.175	.111	.020	.089	.007*	.110	.088
GAF	*r*	.547	.606	.707*	.596	.799*	.553	.606
	*p*	.035	.017	.003*	.019	.000*	.033	.017
GF:SOCIAL	*r*	.493	.582	.691*	.641	.705*	.497	.525
	*p*	.073	.029	.006*	.013	.005*	.070	.054
GAF:GLOBAL	*r*	.478	.561	.719*	.660	.711*	.510	.568
	*p*	.084	.037	.004*	.01	.004*	.062	.034

*Note*: *Significant correlations after applying Bonferroni correction (*p* < .0083).

Abbreviations: GAF, Global Functioning Scale; GF: Role, Global Functioning; GF: Social, Global Functioning: Social; HAMD, Hamilton Depression Rating Scale; PANSS, Positive and Negative Syndrome Scale; SANS, Scale for the Assessment of Negative Symptoms.

**Figure 3:**
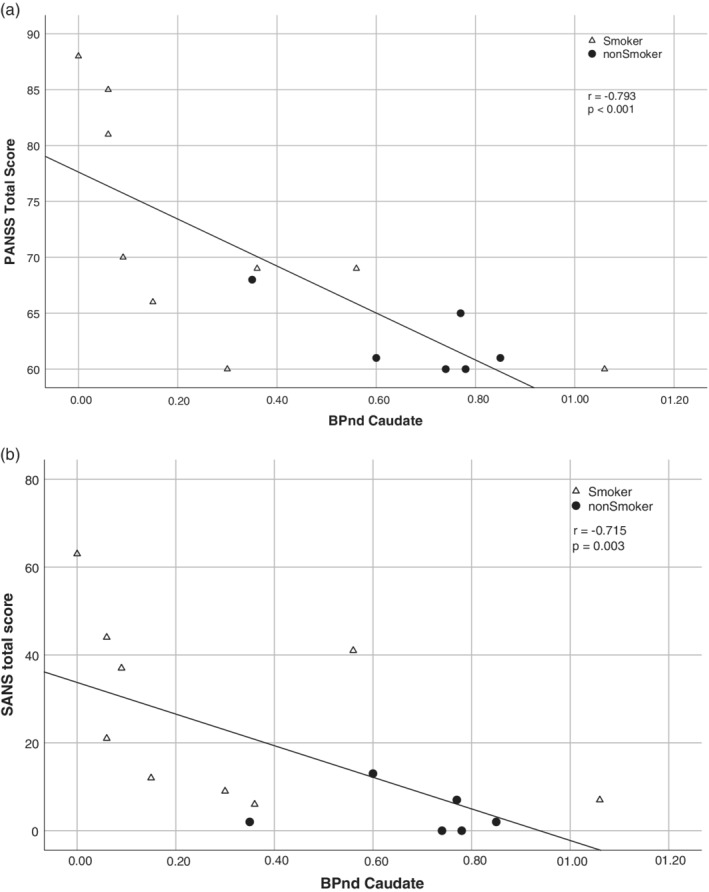
Association between the (a) PANSS total score and mGluR5 receptor binding, and the (b) SANS total score and mGluR5 receptor binding in the caudate in schizophrenia patients

The PANSS total score correlated significantly with BP_ND_ in almost all the observed regions (except the left prefrontal gyrus and putamen, with respect to the Bonferroni corrected significance level 0.0083). Figure 3a shows the caudate as an example. The PANSS general score correlated significantly with BP_ND_ in the right prefrontal gyrus, left temporal, caudate and the inferior frontal gyrus (*r* values between −0.665 and −0.803; *p* ≤ .0083). The PANSS negative score correlated significantly with BP_ND_ in the left temporal and caudate (*r* values −.714 and −.679, respectively; *p* ≤ .0083 both). The PANSS positive score did not correlate significantly with BP_ND_. The SANS global score only correlated significantly with BP_ND_ in the caudate (Figure 3b).

**Figure 4:**
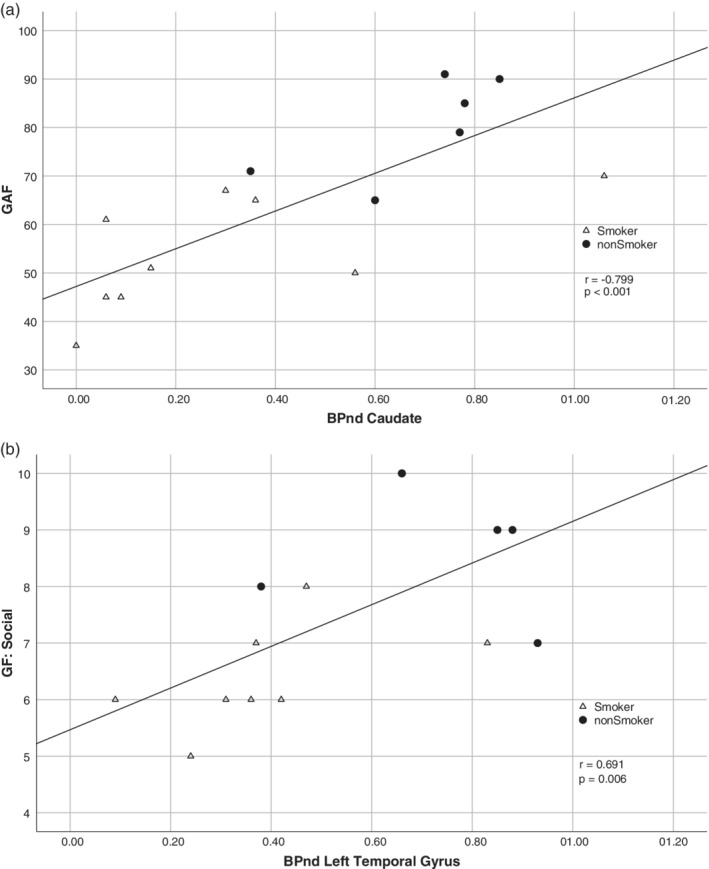
Association between (a) Global Functioning Scale (GAF) and mGluR5 receptor binding in the caudate, and (b) Global Functioning: Social Scale (GF: Social) and mGluR5 receptor binding in the left temporal cortex in schizophrenia patients

With respect to the single subscales, the SANS‐affective flattening subscale correlated with BP_ND_ in the left temporal and inferior frontal gyrus (*r* values: −.669 and −.660, respectively). Furthermore, the SANS‐Alogia subscale correlated significantly with BP_ND_ in the left temporal and caudate regions (*r* values −.690 and −.708, respectively). The SANS Avolition/apathy subscale correlated significantly with BP_ND_ in almost of all regions (*r* values between −.687 and −.792), except in the left prefrontal gyrus. The SANS‐Anhedonia subscale did not correlate with the analyzed regions, as was also noticed for the SANS attentional impairment subscale (*p* > .0083 for all). The HAMD score correlated significantly with BP_ND_ in the caudate (*r* = −.660). The GAF correlated significantly with BP_ND_ in the left temporal (*r* = .710) and caudate (*r* = −.799; Figure 4a). The GF‐social functioning score showed also significant correlations with BP_ND_ values in the left temporal and caudate (*r* values: .690 and .705, respectively). Finally, a similar high correlation can be found with the GF‐global functioning score in the left temporal (Figure 4b) and caudate (*r* values: .719 and .711, respectively).

### 3.4 Neurocognitive performance

All results relating to the neurocognitive tests are shown in Table 5. After applying the Bonferroni correction, all correlations with a *p* value ≤.0083 were considered significant.

**Table 5:** Results of the neurocognitive tests performed by schizophrenia patients and healthy controls and comparison between the groups (Student's *t*‐test)

	Patients	Healthy controls	Patients versus healthy controls (*t*‐test) *p* values
TMT(A) (s)	39.35 ± 15.74	28.80 ± 9.61	.039
TMT(B) (s)	97.06 ± 45.42	61.75 ± 25.81	.022
TMT(B)–TMT(A)	59.19 ± 35.43	32.37 ± 21.07	.031
TMT(B)/TMT(A)	2.62 ± 0.94	2.16 ± 0.67	.172
CT (sum score)	59.67 ± 17.34	68.50 ± 14.75	.150
LNS (sum score)	18.60 ± 2.23	20.93 ± 1.77	.005*
LNS (longest sequence)	4.80 ± 0.94	5.64 ± 0.75	.013
CBT—forward	7.93 ± 1.28	9.00 ± 2.04	.100
CBT—backward	8.13 ± 3.89	8.50 ± 1.61	.750
RVDLT valid recognitions	59.53 ± 20.33	81.21 ± 13.63	.002*
RVDLT—errors	10.00 ± 6.19	7.14 ± 6.73	.240
z‐composite score	−0.34 ± 0.70	0.36 ± 0.45	.003*

*Notes*: Values are given as mean ± *SD*. *Significant results after Bonferroni correction (*p* < .0083).

Abbreviations: CBT, Corsi block‐tapping task; CT, Number‐symbol coding task; LNS, Letter–Number Span; RVDLT, Rey Visual Design Learning Test; TMT(A), Trail‐Making Test (A); TMT(B), Trail‐Making Test (B).

### 3.5 Performance speed, mental flexibility, and executive functions

Schizophrenia patients (SZ) in both TMT subtests required a longer time to perform the tasks than healthy controls (HC). However, the results were not significant after Bonferroni correction (*p* > .0083 for all). In the coding task, there were no significant differences between SZ and HC (*p* = .15).

### 3.6 Working memory

In the letter‐number span (LNS), the SZ patients reached a significantly lower sum score than the healthy controls (*p* = .005), but not a significant maximal sequence length (*p* = .013). The performance of the patients in the Corsi block‐tapping task (forward and backward) was not significantly different from the HC (*p* = .10 and *p* = .75, respectively).

### 3.7 Visual memory

In the RVDLT, patients performed worse than the healthy controls, showing a significantly lower number of valid recognitions (*p* = .002), but a nonsignificant number of errors (*p* = .240).

### 3.8 Composite z‐score

Using the procedure described in the methods section, we calculated composite z‐scores for all cognitive tasks and found them to be significantly lower in patients (*p* = .003).

### 3.9 Role of smoking for neurocognitive performance

The results of the neurocognitive tests are depicted separately for smoking status in Table 6. The comparison between the subgroups was performed using the nonparametric Wilcoxon test. In the patient group, smokers and nonsmoker subjects differed significantly only in the coding task (*p* = .003). No significant differences were found for any of the tasks in the healthy controls group (*p* > .0083).

**Table 6:** Results of the neurocognitive tests performed by schizophrenia patients and healthy controls, separately depicted for smokers and nonsmokers

	Patients			Healthy controls		
	Smoker (*N* = 9)	Nonsmoker (*N* = 6)	Smoker versus nonsmoker (Wilcoxon‐test; *p*‐value)	Smoker (*N* = 8)	Nonsmoker (*N* = 7)	Smoker versus nonsmoker (Wilcoxon‐test; *p*‐value)
TMT(A) (s)	47.04 ± 14.9	27.80 ± 8.44	.026	34.56 ± 9.13	30.07 ± 6.33	.038
TMT(B) (s)	121.00 ± 42.95	65.15 ± 25.35	.029	78.60 ± 23.46	49.70 ± 21.17	.073
TMT(B)–TMT(A)	75.59 ± 35.75	37.33 ± 21.68	.059	40.40 ± 22.61	26.64 ± 19.51	.343
TMT(B)/TMT(A)	2.76 ± 0.98	2.43 ± 0.95	.491	2.09 ± 0.53	2.20 ± 0.79	.900
CT (sum score)	50.33 ± 13.03	73.67 ± 13.35	.003*	63.57 ± 9.98	73.43 ± 17.74	.165
LNS (sum score)	18.00 ± 2.00	19.50 ± 2.43	.272	20.57 ± 1.98	21.29 ± 1.60	.456
LNS (longest sequence)	4.56 ± 0.89	5.17 ± 0.98	.272	5.43 ± 0.79	5.86 ± 0.69	.259
CBT—forwards	7.56 ± 1.01	8.50 ± 1.52	.181	9.14 ± 2.27	8.86 ± 1.95	.902
CBT—backwards	8.00 ± 4.87	8.30 ± 2.1	.388	8.86 ± 0.90	8.14 ± 2.11	.259
RVDLT valid recognitions	55.67 ± 20.89	65.33 ± 19.78	.388	79.57 ± 11.99	82.86 ± 15.89	.710
RVDLT—errors	10.11 ± 7.01	9.83 ± 5.35	.955	7.43 ± 6.19	6.86 ± 7.73	.710
z‐composite score	−0.64 ± 0.64	0.10 ± 0.56	.036	0.22 ± 0.38	0.51 ± 0.49	.165

*Notes*: In addition, a comparison between smokers and nonsmokers (Wilcoxon test). Values are given as mean ± *SD*, and *significant result after Bonferroni correction (*p* < .0083).

Abbreviations: CBT, Corsi block‐tapping task; CT, Number‐symbol coding task; LNS, Letter–Number Span (LNS); RVDLT, Rey Visual Design Learning Test; TMT(A), Trail‐Making Test (A); TMT(B), Trail‐Making Test (B).

### 3.10 Relationship between neurocognitive performance and mGluR5 BP_ND_ values

The relationship between the neurocognitive performance and mGluR5 binding was examined separately for each group by calculating a Spearman's rank correlation coefficient between the scores reached in the neurocognitive tests and BP_ND_ values in all observed brain regions. All correlations for the patient group are shown in Table 7.

**Table 7:** Relationship between the neurocognitive performance and mGluR5 receptor binding exploratory examined by calculating Spearman's rank correlation coefficient between scores reached in the neurocognitive tests and BP_ND_ for the seven observed brain regions in the patient group

		Left prefrontal gyrus	Right prefrontal Gyrus	Left temporal	Right temporal	Caudate	Putamen	Inferior frontal gyrus
Subjects	*N*	15	15	15	15	15	15	15
TMT(A) (s)	*r*	−.408	−.445	−.544	−.423	−.571	−.449	−.486
	*p*	.131	.096	.036	.117	.026	.093	.066
CT	*r*	.531	.550	.750*	.615	.665*	.517	.576
	*p*	.042	.033	.001*	.015	.007*	.049	.025
LNS (sum score)	*r*	−.051	−.114	.082	.028	−.022	−.069	−.035
	*p*	.857	.685	.772	.921	.939	.807	.903
LNS (longest sequence)	*r*	−.090	−.145	.016	−.081	.039	−.125	−.065
	*p*	.749	.607	.955	.773	.891	.658	.817
CBT—forward	*r*	.164	.190	.311	.216	.222	.170	.221
	*p*	.559	.497	.259	.439	.426	.545	.429
CBT—backward	*r*	.283	.322	.245	.191	.524	.255	.330
	*p*	.307	.242	.378	.495	.045	.359	.230
RVDLT valid recognitions	*r*	.193	.209	.164	.079	.363	.241	.220
	*p*	.490	.454	.560	.780	.183	.386	.430
RVDLT—errors	*r*	−.048	−.074	.026	.084	−.204	−.097	−.015
	*p*	.866	.794	.927	.765	.466	.731	.957
Z‐composite score	*r*	.288	.307	.433	.300	.500	.321	.322
	*p*	.298	.265	.107	.277	.057	.243	.242
TMT(B) (s)	*r*	−.203	−.279	−.392	−.398	−.341	−.385	.304
	*p*	.487	.334	.166	.158	.233	.175	.291
TMT(B)–TMT(A)	*r*	−.161	−.231	−.315	−.343	−.271	−.358	−.264
	*p*	.583	.427	.273	.230	.349	.208	.361
TMT(B)/TMT(A)	*r*	.242	.169	.103	.075	.084	.055	.123
	*p*	.404	.563	.725	.799	.776	.852	.674

*Note*: *Significant correlations after Bonferroni correction (*p* < .0083).

Abbreviations: CBT, Corsi block‐tapping task; CT, Number‐symbol coding task; LNS, Letter–Number Span (LNS); RVDLT, Rey Visual Design Learning Test; TMT(A), Trail‐Making Test (A); TMT(B), Trail‐Making Test (B).

**Figure 5:**
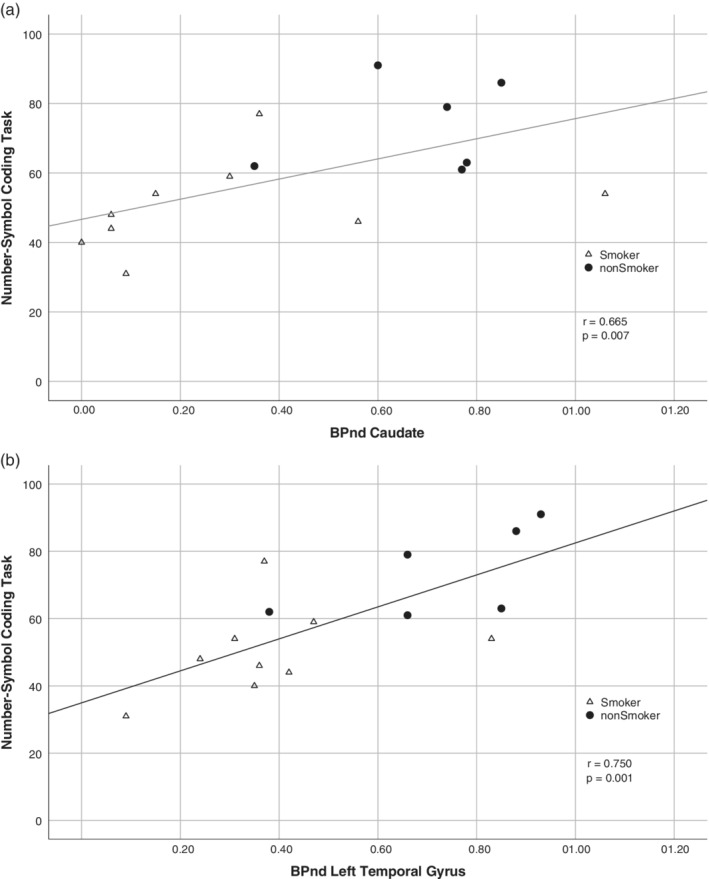
Association between the neurocognitive performance in (a) Number‐Symbol Coding Task and mGluR5 receptor binding in caudate, and (b) Number‐Symbol Coding Task and mGluR5 receptor binding in the left temporal cortex in schizophrenia patients

After applying Bonferroni correction, only correlations between the coding task performance and the caudate (Figure 5a), and the left temporal (Figure 5b) remained significant (*r* values: .665 and .750, respective; *p* < .0083 for both). No significant correlations were found in the HC group.

## 4 DISCUSSION

This exploratory PET study aimed to contribute to a better understanding of the role of glutamatergic neurotransmission in psychopathology and cognitive impairments in schizophrenia. Such associations have been indicated in previous animal studies, but up to now, they have not been sufficiently proven in patients suffering from schizophrenia. Thus, we tested for correlations between the mGluR5 receptor BP_ND_ and psychopathology and cognitive performance in selected striatal and frontotemporal cortex regions using [^11^C]ABP688 in two groups: One comprising 15 male schizophrenia patients (which all were chronically ill and on stable medication with second‐generation antipsychotics) and one comprising 15 demographically matched healthy volunteers.

Our study revealed the following four main findings.

First, higher mGluR5 BP_ND_ values were associated with lower general symptom levels (in particular negative and depressive symptoms as measured with the PANSS, SANS and HAMD scores), as well as with higher levels of global functioning scores (GAF, GF).

Second, patients with higher mGluR5 BP_ND_ values showed better cognitive performance. Such an association was not found in healthy controls.

Third, no significant differences were found in mGluR5 BP_ND_ between healthy subjects and patients with schizophrenia. The numerically higher values in healthy controls were not statistically significant. Smoking status showed a strong effect in both groups, suggesting a close association between tobacco/nicotine use and the glutamatergic system that is particularly pronounced in schizophrenia.

Finally, in the patient group, being an active smoker was a common denominator for individuals with more pronounced negative symptoms, worse global functioning, poorer cognitive performance as well as lower mGluR5 BP_ND_. This finding raises the question as to whether glutamatergic dysfunction, particularly associated with a downregulation of mGluR5, may be a link between disease severity and comorbid nicotine addiction in schizophrenia.

Our results are in line with the current conception of the glutamate hypothesis of schizophrenia, which emphasizes the main role of the aberrant glutamate receptors in this disease (Hammond et al., 2014). Up to now, the most consistent findings have been reported for NMDA‐R, suggesting that NMDA‐R dysfunction represents a final common pathway leading from pathogenesis to symptoms in schizophrenia (Javitt, 2010). Comparatively, relatively little is known about the involvement of mGluR5 receptors in the pathophysiology of schizophrenia. A considerable number of studies have emphasized the apparently significant role of mGluR5 signaling in schizophrenia and its interaction with abnormal NMDA‐R activity in this disease (Wang et al., 2018). Furthermore, the importance of mGluR5 for schizophrenia has been confirmed in genetic (Kordi‐Tamandani, Dahmardeh, & Torkamanzehi, 2013; The Network and Pathway Analysis Subgroup of Psychiatric Genomics Consortium, 2015) and animal studies (as already outlined in the introduction). Additionally, Wang et al. (2018) recently provided the first direct evidence for mGluR5 hypoactivity in dorsolateral prefrontal cortex tissue taken post‐mortem from schizophrenia patients, although several previous investigations could not find altered mGluR5 levels postmortem in schizophrenia (Matosin, Frank, Deng, Huang, & Newell, 2013) or psychotic depression (Matosin et al., 2014). Furthermore, the concept of mGluR5 dysregulation in schizophrenia is supported by several other investigations, including various disturbed mechanisms underlying transcription and translation of mGluR5, as well as alterations to mGluR5 trafficking, distributions, phosphorylation, and protein–protein interactions (Matosin et al., 2017). If it is accepted that mGluR5 hypofunction is an underlying factor in the symptomatology of schizophrenia, higher mGluR5 receptor availability may mitigate the symptoms. Our analysis supports this concept as it reveals lower general symptom levels (in particular, negative and depressive symptoms as measured with PANSS, SANS, and HAMD scores) and higher levels of global functioning associated with higher mGluR5 BP_ND_.

In our study, higher mGluR5 receptor binding was beneficial in terms of lower SANS and HAMD scores. In line with this finding, Thiebes et al. (2017) reported a significant increase in negative symptoms after antagonizing NMDA‐R with ketamine in healthy volunteers. This was also tightly associated with deficits in mismatch negativity (MMN), which is known to be robustly reduced in schizophrenia (Salisbury, Shenton, Griggs, Bonner‐Jackson, & McCarley, 2002). Thus, further investigation of ways to increase mGluR5 associated glutamatergic functionality (and consecutively the NMDA‐receptor activity) may be a promising approach for the treatment of negative symptoms in schizophrenia.

Our second major finding suggests the involvement of mGluR5 receptors in cognition in schizophrenia patients. Generally, numerous previous investigations have indicated that glutamatergic synapses in the brain, responsible for fast excitatory neurotransmission, play a critical role in a broad range of cognitive functions. Thereby, the majority of studies support the key role of NMDA‐R in cognition (Dauvermann, Lee, & Dawson, 2017). Nevertheless, pharmacological studies using NMDA‐R modulators, such as glycine, d‐serine, or d‐cycloserine, in order to improve cognitive performance in humans, have been less persuasive up to now (Iwata et al., 2015). Thus, during recent years, the focus of interest for some drug development studies has moved to the mGluR5 as a target of positive allosteric modulators (PAM), as referred to in the introduction. In terms of this connection, Matosin et al. (2013) reported unaltered allosteric binding site and mGluR5 protein levels in schizophrenia pathology, suggesting that the binding potential of mGluR5 PAMs will not be affected in patients with schizophrenia, qualifying them as suitable therapeutic agents. Our finding of an association between cognition and the mGluR5 supports this approach and encourages the idea of further development of such agents in order to target cognitive impairments in schizophrenia.

Interestingly, the association between higher mGluR5 receptor binding and better cognitive performance primarily concerned the coding task performance and the mGluR5 receptor binding in the left temporal cortex and caudate (bilateral). Generally, a slower speed of information processing is considered to be one of the central features of cognitive impairment in schizophrenia (Rodriguez‐Sanchez et al., 2007). Several investigations support the contention that global cognitive dysfunction in schizophrenia may be determined, to some extent, by impaired processing speed (Andersen et al., 2013). Indeed, impairments in coding tasks are often significantly more pronounced than impairments in measures from memory, attention, reasoning, and problem solving, and working memory domains in schizophrenia (Andersen et al., 2013). Coding task performance deficits have usually been associated with altered function in the parietal, temporal, and frontal regions (Turken et al., 2008). In particular, a strong correlation has been reported between impaired cognition and volume decrement or structural abnormalities in some temporal lobe subregions in schizophrenia (Roalf et al., 2017). Furthermore, it has also been shown that the speed of processing is associated with frontotemporal white matter abnormalities (Rigucci et al., 2013). In our study, higher mGluR5 receptor binding in the left (but not in the right) temporal region was beneficial for speed of information processing. This asymmetry is in accordance with the long‐standing notion that schizophrenia is characterized by a disruption of the normal pattern of emerging cerebral asymmetry, which primarily affects development in the left hemisphere and leads to more prominent left hemisphere dysfunction (Crow et al., 1989). Thus, our findings emphasize the importance of glutamatergic neurotransmission in the left temporal region for information processing in schizophrenia.

Furthermore, the speed of information processing was associated with mGluR5 BP_ND_ in the caudate. These results complement previous studies describing the importance of striatal dopaminergic neurotransmission for cognition in schizophrenia. Reeves et al. (2005) reported a significant association between striatal D_2_‐dopamine receptor availability and spatial planning. In our own previous work, we were also able to demonstrate an association between better executive functional and higher striatal D_2_‐dopamine receptor availability in schizophrenia patients but not in healthy volunteers (Veselinovic et al., 2013). With the current results, we provide additional confirmation of the importance of striatal neurotransmission for cognition in schizophrenia patients, thereby showing that both the dopaminergic system and also the glutamatergic system seem to be involved in the underlying processes.

However, it is not possible to speculate about the actual mechanisms behind the potentially pro‐cognitive effects conveyed by the mGluR5 receptors based on our data. Although some previous investigators attributed such an effect to the interaction of the mGluR5 receptor with the NMDA‐R, the role of other independent mechanisms is also conceivable (Conn, Lindsley, & Jones, 2009). Several earlier investigations have indicated that mGluR5 form homomers and heteromers with other receptors at the plasma membrane, in a similar way to other G protein‐coupled receptors. Some evidence emphasizes an abnormal function of oligomers, consisting of mGluR5, dopamine D2 (D2R) and adenosine A2A receptors (A2AR‐D2R‐mGluR5; Cabello et al., 2009), as well as D2R‐NMDA hetero‐receptor complexes as major contributors to schizophrenia symptoms (Borroto‐Escuela et al., 2017). According to these findings, altered receptor–receptor interactions in iso‐receptors and hetero‐receptor complexes, as well as changes in their balance with each other, may be a basis for altered neuromodulation and dysfunction of the brain circuits, resulting in deficits in learning, memory, emotions, and motivation in depression, addiction, and schizophrenia (Borroto‐Escuela et al., 2017). Consequently, these hetero‐receptors may provide targets for a new therapeutic approach for some neuropsychiatric disorders (Fuxe et al., 2003; Popoli et al., 2001).

In our study, we were unable to demonstrate a significant difference regarding the mGluR5 availability between schizophrenia patients and healthy volunteers. We observed a trend of lower BP_ND_ in all analyzed regions in the patient group, although without a statistical significance. To the best of our knowledge, the only previous similar in vivo study using the same radioligand, also reported an absence of difference in mGluR5 DVR between patients and controls (Akkus et al., 2017). Furthermore, in this study, smoking status was associated with marked global reductions in mGluR5 availability in both groups. In our study, we confirm these findings. Acknowledging the very high prevalence of smoking (more than 60%) in subjects with schizophrenia (Chapman, Ragg, & McGeechan, 2009) and their greater difficulties in quitting smoking (Wing, Wass, Soh, & George, 2012), a more precise understanding of the common neurobiological basis of schizophrenia and nicotine dependence is of particular importance. Several investigations have emphasized the potential usage of tobacco by schizophrenia patients for self‐medication purposes in order to alleviate specific symptoms (Lucatch, Lowe, Clark, Kozak, & George, 2018), particularly negative symptoms and dysfunctional reward circuits (Peechatka, Whitton, Farmer, Pizzagalli, & Janes, 2015). However, investigations in this field have yielded mixed findings. Several authors have reported comparable or even worse cognition within chronic smokers with schizophrenia (Hickling et al., 2018). Moreover, studies that carefully controlled for the time since the last cigarette have found that smoking produces cognitive deficits in schizophrenia, particularly in working memory, visual learning, and attention (Reed, Harris, & Olincy, 2016). Thus, opposing to the earlier self‐medication hypothesis, those investigations indicate a bidirectional relationship between smoking and psychosis wherein cigarette smoking may be causally related to a risk of psychosis, possibly via a shared genetic liability (Quigley & MacCabe, 2019). In our study, smoking habit was a common denominator for more pronounced negative and depressive symptoms, poorer functioning level, lower cognitive performance, and lower mGluR5 receptor availability. This finding emphasizes a possible mediating role of glutamate, and in particular mGluR5, as a link between schizophrenia and smoking.

Several methodological limitations should be kept in mind when interpreting our results. One of the main limitations of the study may be the joint consideration of smokers and nonsmokers as one group during the investigation of the associations between BP_ND_ and psychopathology and cognition. However, the role of smoking for all observed parameters has been discussed extensively. Despite this possible drawback, this integrative approach highlights the potential common basis of symptom severity and nicotine addiction, which could be anchored in glutamatergic dysfunctions.

A second potential limitation of the study is that the relatively small sample size prohibits a broader generalization of our findings. This is a common problem in PET studies due to the high requirements for patients suitable for inclusion. A particular challenge for studies using [^11^C]ABP688 is the necessity to account for the smoking status as a specific influence factor and to ensure a perfect matching regarding this confounder. However, as the effect of smoking is so grave and has already been replicated across studies, an increase in sample size would probably not change our results significantly.

A third potential limitation of our study relates to the illness duration of the patients included in the sample (from 8 to 32 years), and thus different illness stages. However, one pre‐request for inclusion in the study was a pre‐existing clinical attribution as a chronic patient. In particular, first‐episode patients were not included. Furthermore, our analysis revealed an absence of significant correlations between the BP_ND_ and illness duration in all regions. In addition, in order to reduce the sample heterogeneity, only male subjects were included and patients with comorbid consumption of illegal drugs (incl. cannabis) were excluded. One more variability factor previously described for PET studies using [^11^C]ABP688—the daytime variability (DeLorenzo et al., 2017)—was eliminated by scheduling all PET data acquisitions at the same time of the day (late morning) for all subjects in this study. Thus, our study design ensured the elimination of several confounding factors (gender diversity, cannabis influence, study design based on mGluR5/glutamate system variations) that were not considered in some other previous studies investigating the glutamatergic system in schizophrenia.

Fourth, although all patients in our study were treated with second‐generation antipsychotics in common doses, we did not control for the type and duration of previous medication. Due to the lack of a central/lifetime medical register, this information was not available in a reliable form in the majority of the cases. Nevertheless, we could not find any association between the medication (e.g., CPZ equivalents) and mGluR5 BP_ND_.

## 5 CONCLUSION

Our study revealed no significant differences (10% on average in all evaluated brain regions in nonsmokers subjects) between schizophrenia patients and healthy controls regarding mGluR5 receptor availability. However, low mGluR5 receptor availability was identified as a common factor associated with more pronounced negative and depressive symptoms, lower levels of global functioning and poorer cognitive performance in a sample of 15 male, chronically ill, stable schizophrenia patients. The findings were strongly associated with the smoking status of the subjects. The causal direction of this linkage remains unclear and requires further investigation.

In particular, future mGluR5 imaging studies should, ideally, include unmedicated, nonsmoking patients from the first onset of the disease. However, despite some possible limitations, our study provides a new perspective on the complex relationship between tobacco smoking and schizophrenia and points toward the possible role of glutamatergic neurotransmission—particularly the metabotropic glutamate receptors type 5—in this interrelation. Further investigation may be a promising approach for a better understanding of the common aspects of inclination toward tobacco smoking and psychosis.

## CONFLICT OF INTEREST

No potential conflict of interest relevant to this article was reported.

## AUTHOR CONTRIBUTIONS

C.R.B.: PET bolus‐infusion protocol optimization, data acquisition, PET imaging and plasma data analysis, image processing (BP_ND_), statistical design and analysis, manuscript writing (52%), correction, and revision. T.V.: Psychopathology scores and BP_ND_ correlation analysis, statistical analysis, manuscript writing (48%), correction and revision. R.R.: Data acquisition and revision of the manuscript. J.M.: PET bolus‐infusion study design, data acquisition, and revision of the manuscript. L.O.: Volunteer screening and data acquisition. A.R.: Volunteer screening and revision of the manuscript. S.R.: Data acquisition and revision of the manuscript. K.H.: Study design and revision of the manuscript. W.K.: Study design and revision of the manuscript. C.W.: Study design and revision of the manuscript. E.R.K.: MR‐PET attenuation correction, revision, and correction of the manuscript. L.T.: MR‐PET hardware and data acquisition. J.S.: PET reconstruction and head motion correction methodology. F.B.: MR‐PET hardware integration and study design. B.N.: Revision of the manuscript. J.E.: Revision and corrections of the manuscript. H.H.: PET study design discussions, revision, and corrections of the manuscript. K.‐J.L.: Revision of the manuscript. N.J.S.: MR‐PET hardware and revision of the manuscript. C.L.: Study design and setup, data analysis revision, manuscript corrections, and revision. I.N.: Study design and setup, approval ethics and BfS, and revision of the manuscript.

## Data Availability

Raw data were generated at Forschungszentrum Jülich and RWTH Uniklinik Aachen. Derived data supporting the findings of this study are available from the corresponding and first authors C.R.B and T.V. on request.
